# What Dietary Vitamins and Minerals Might Be Protective against Parkinson’s Disease?

**DOI:** 10.3390/brainsci13071119

**Published:** 2023-07-24

**Authors:** Mohammad Alizadeh, Sorayya Kheirouri, Majid Keramati

**Affiliations:** 1 Department of Nutrition, Faculty of Nutrition and Food Sciences, Tabriz University of Medical Sciences, Tabriz 5166614711, Iran; mdalizadeh@tbzmed.ac.ir (M.A.); majidkeramati87@gmail.com (M.K.); 2 Nutrition Research Center, Tabriz University of Medical Sciences, Tabriz 5166614711, Iran

**Keywords:** Parkinson’s disease, dietary intake, vitamins, minerals, lycopene, thiamine, vitamin B6, vitamin B12, pantothenic acid, magnesium, zinc, manganese, selenium, chromium, phosphorus, α-tocopherol, potassium

## Abstract

Background and Objective: Dietary constituents may affect the progression of Parkinson’s disease (PD). This study aimed to assess the contribution of dietary intake of vitamins and minerals to the severity, motor and non-motor symptoms, and risk of PD. Methods: In this case-control study, 120 patients with PD and 50 healthy participants participated. Dietary intake of vitamins and minerals was determined using a 147-item food frequency questionnaire. The severity of PD was determined by the Unified Parkinson’s Disease Rating Scale (UPDRS). Results: Patients with PD had lower intake of several vitamins and minerals including lycopene, thiamine, vitamin B6, vitamin B12, pantothenic acid, magnesium, zinc, manganese, selenium, chromium, and phosphorus, but had higher intake of α-tocopherol. High dietary intake of vitamin A, α-carotene, β-cryptoxanthin, vitamin C, and α-tocopherol were correlated with increased odds of PD. High intake of lycopene, thiamin, vitamin B6, pantothenic acid, magnesium, zinc, manganese, chromium, and phosphorous correlated with reduced odds of PD. The predictive power of α-tocopherol concerning the risk of PD was stronger relative to other vitamins. Dietary intake of pantothenic acid was negatively correlated with PD severity and symptoms of motor examination and complication. The severity and motor symptoms of PD were also negatively correlated with β-carotene, vitamin C, riboflavin, vitamin B6, and biotin intake. The UPDRS total score and motor symptoms in PD patients were negatively correlated with phosphorus, magnesium, zinc, manganese, and chromium, and strongly with potassium intake. Conclusion: The findings indicate that adequate dietary intake of vitamins and minerals may have a preventive effect on developing PD and progression of motor decline.

## 1. Introduction

Parkinson’s disease (PD) is an age-related and progressive neurological disorder that mainly affects the motor system. The loss of nerve cells in a brain area named the substantia nigra, responsible for producing dopamine, is known as the cause of the disease. Tremor, rigidity, slowness of movement, balance problems, and difficulty with coordination and motor functions are all common early symptoms of the disease. As the disease progresses, non-motor symptoms including autonomic dysfunction, neuropsychiatric problems, and sensory and sleep difficulties are commonly emerging [1]. Globally, 6.1 million individuals had PD in 2016, compared with 2.5 million in 1990, and the global burden of the disease has more than doubled [2]; 3.2 million DALYs and 211,296 deaths were attributed to PD in 2016 [2].

The exact pathogenic pathway of PD is unknown, however, according to the evidence, abnormal accumulation of α-synuclein protein, decreased levels of prohibitin protein in substantia nigra, and neuroinflammation may contribute to PD pathogenesis [3,4,5,6]. The aggregation of α-synuclein is related to the dysfunctionality and degeneration of neurons in PD [6]. Reduced levels of prohibitin are led to dopaminergic cell death in substantia nigra [3]. A higher ratio of neutrophil-to-lymphocyte and platelet-to-lymphocyte, as non-specific parameters of inflammation, in α-synucleinopathies has been reported in PD patients [4,5]. Several other factors including age, genetic (such as mutation in the LRRK2 gene), exercise, and environmental factors (such as diet and exposure to toxins) may also contribute to the etiology of the disease [7,8,9].

Lifestyle and environmental factors such as diet may have the potential in developing or management of PD. Dietary constituents may affect the progression of the disease and some certain food ingredients can be protective against the risk and symptoms of the disease [10]. For instance, food items such as fresh vegetables and fruits were associated with a reduced rate of PD progression, but canned fruits and vegetables with a more rapid progression of PD [10]. Hughes et al. reported that the type of dairy products could affect PD risk. Results of two large prospective cohort studies showed that increased consumption of low-fat dairy products increased the risk of PD, but greater intake of high-fat dairy products reduced it [11]. Agarwal et al. reported that several nutrients including vitamin E, vitamin C, and carotenoids may be useful in reducing the risk of PD [12]. In addition, an increased risk of malnutrition can increase motor symptom severity in people with PD [13]. Moreover, dietary patterns [14,15] and whole diet characteristics such as anti-inflammatory and antioxidant properties may also influence PD risk [16].

Since diet may contribute to PD risk and motor decline, the evaluation of the dietary intake of the patients is important to determine the role of micronutrient inadequacies in the etiology of the disease. Therefore, this study aimed to assess the contribution of dietary intake of vitamins and minerals in the severity, motor and non-motor symptoms, and risk of PD.

## 2. Materials and Methods

### 2.1. Study Population

This case-control study was performed on 120 patients with Parkinson’s disease and 50 healthy individuals in Isfahan City, Iran. Samples were collected from a clinical department using a convenience sampling method. Inclusion criteria were age 40–80 years and diagnosis and confirmation of PD by a neurologist based on the Hoehn and Yahr (H & Y) Staging Scale [17]. Exclusion criteria were receiving enteral and parenteral nutritional support, admission to hospital wards, having any gastrointestinal disease that interferes with the digestion and absorption of food, and using any vitamin or mineral supplement. The inclusion and exclusion criteria of the control group were the same as the PD group except that they do not have any diseases. Participants of the two groups were matched by age and gender. Informed consent was obtained from each participant. The study was approved by the ethics committee of Tabriz University of Medical Sciences.

### 2.2. Assessment of Dietary Intake

The dietary intake of participants was assessed using a 147-item semi-quantitative food frequency questionnaire (FFQ). All the questionnaires were completed by a nutritionist. The FFQ contained a list of foods with serving sizes that are commonly consumed by Iranians. Participants were asked to report the frequency of each food item consumed in the past year on a daily, weekly, or monthly basis. The quantity of intake for each food parameter was asked as the household or usual amounts such as 1 slice, 1 tablespoon (15 mL), or 1 cup (250 mL), indicating 1 standard serving. The intake of each food item was converted to grams per day by multiplying the frequency of intake, the quantity of intake, and the standard serving size of each food item. Then, the daily grams of intake for each food item were summed to obtain the daily intake of each food group. The modified Nutritionist software version 4 (First Data Bank, San Bruno, CA, USA) was used to obtain the intake of vitamins (including vitamin A, α-carotene, β-carotene, lutein, β-cryptoxanthin, lycopene, vitamin C, vitamin D, vitamin E, α-tocopherol, thiamine, riboflavin, niacin, vitamin B6, folate, vitamin B12, biotin, pantothenic acid, and vitamin K) and minerals (including iron, magnesium, zinc, copper, manganese, selenium, chromium, calcium, phosphorus, fluorine, and potassium). The reliability and validity of the Persian version of this questionnaire have already been confirmed [18].

### 2.3. Assessment of Anthropometric Parameters

The height of participants was measured using a tape meter in a standing position without shoes, to the nearest 0.5 cm. The weight was measured using a SECA scale with minimal coverage and without shoes and with an accuracy of 100 g. BMI was calculated by dividing weight (kg) by height squared (m).

### 2.4. Severity of PD

The severity of PD was assessed by an expert neurologist. All aspects of PD were evaluated by the Movement Disorder Society-sponsored Unified Parkinson’s Disease Rating Scale (MDS-UPDRS). MDS-UPDRS includes 65 items and a range of 0 to 272 scores in 4 parts: Part I, non-motor aspects of experiences of daily living (13 items); Part II, motor aspects of experiences of daily living (13 items); Part III, motor examination (33 items), and Part IV, motor complications (6 items).

### 2.5. Statistical Analysis

Findings were analyzed using IBM SPSS software version 23 (SPSS, Inc., Chicago, IL, USA). Descriptive evidence and the Kolmogorov–Smirnov test were used to investigate the distribution of variables (normal or non-normal). An independent *t*-test was used to compare normal data, and a Mann–Whitney test was used for non-normal data between the two studied groups. Participants were stratified into tertiles according to dietary intake of vitamins and minerals. A binary logistic test was used to determine the odds of PD across the tertiles. Linear regression was used to examine the association between variables. Age, gender, BMI, total energy intake, smoking, diabetes, hypertension, thyroid disorder, cardiovascular diseases, and medications were used as confounding factors in the analysis. The relative abilities of various vitamins and minerals were compared using receiver operator characteristics (ROC) to predict PD. A *p*-value less than 0.05 was considered significant for all statistical assessments.

## 3. Results

The demographic characteristics of the participants are shown in Table 1; 65.8% of patients and 66% of healthy participants were men. There was no significant difference between the two groups in age, sex, and BMI (*p* > 0.05). The mean ± SD of total UPDRS was 46.2 ± 25.2 in the patient’s group. Smoking status was significantly lower in patients (9.2%) compared with healthy subjects (24.0%) (*p* = 0.01).

### 3.1. Dietary Intake of Vitamins and Minerals

As shown in Table 2, dietary intake of lycopene (*p* = 0.03), thiamin (*p* = 0.03), vitamin B6 (*p* = 0.02), vitamin B12 (*p* = 0.03), and pantothenic acid (*p* = 0.004) were significantly lower in patients with PD compared with healthy subjects. Dietary intake of α-tocopherol was higher in PD patients compared with healthy subjects (*p* = 0.05). As shown in Table 3, patients with PD had a significantly lower dietary intake of magnesium (*p* = 0.02), zinc (*p* = 0.02), manganese (*p* = 0.005), selenium (*p* = 0.02), chromium (*p* = 0.01), and phosphorus (*p* = 0.02) compared with healthy subjects.

As shown in Supplementary Table S1, in total participants, men had a significantly lower dietary intake of vitamin A (*p* = 0.05), β-carotene (*p* = 0.03), and vitamin C (*p* = 0.047), but higher thiamin (*p* < 0.001), niacin (*p* < 0.001), folate (*p* < 0.001), and biotin (*p* = 0.05) intake compared with women. In patients with PD, men had higher dietary intake of thiamin (*p* = 0.001), niacin (*p* = 0.001), folate (*p* = 0.01), and biotin (*p* = 0.04) compared with women. In healthy subjects, men had a significantly lower dietary intake of vitamin A (*p* = 0.04), β-carotene (*p* = 0.04), β-cryptoxanthin (*p* = 0.048), and vitamin C (*p* = 0.03), but higher thiamin (*p* = 0.004), niacin (*p* = 0.002), and folate (*p* = 0.001) intake compared with women.

As shown in Supplementary Table S2, in total participants, dietary intake of iron (*p* < 0.001), magnesium (*p* = 0.005), zinc (*p* = 0.01), copper (*p* < 0.001), manganese (*p* < 0.001), selenium (*p* < 0.001), fluorine (*p* = 0.01), and chromium (*p* < 0.001) were significantly higher in men compared with women. In patients with PD, men had also higher dietary intake of iron (*p* = 0.001), magnesium (*p* = 0.009), zinc (*p* = 0.01), copper (*p* = 0.005), manganese (*p* < 0.001), selenium (*p* < 0.001), and chromium (*p* = 0.002) compared with women. In healthy subjects, men had also significantly higher dietary intake of iron (*p* = 0.001), copper (*p* = 0.01), manganese (*p* < 0.001), selenium (*p* < 0.001), fluorine (*p* = 0.001), and chromium (*p* < 0.001), but lower intake of calcium (*p* = 0.04) compared with women.

### 3.2. Dietary Intake of Vitamins and Minerals and Odds of PD

As shown in Table 4, compared with those in the lowest tertile of dietary vitamin A intake, the odds of PD were 3.57 times (95% CI, 1.05–12.10, *p* = 0.04) more in the highest tertile. Regarding the dietary α-carotene intake, the odds of PD were 3.27 times (95% CI, 1.18–9.04, *p* = 0.02) more in the highest compared with the lowest tertile.

As shown in Table 4, in the highest tertile compared with the lowest tertile, the chance of PD was 3.57 times (95% CI, 1.05–12.10, *p* = 0.04) more for dietary vitamin A intake, 3.27 times (95% CI, 1.18–9.04, *p* = 0.02) for α-carotene, 3.41 times (95% CI, 1.21–9.60, *p* = 0.02) for β-cryptoxanthin, 3.11 times (95% CI, 1.11–8.69, *p* = 0.03) for vitamin C, and 4.31 times (95% CI, 1.51–12.28, *p* = 0.006) for α-tocopherol dietary intake. In the highest tertile, the odds of PD were 76% [OR (95% CI): 0.24 (0.09, 0.67), *p* = 0.006] lower for lycopene, 86% [OR (95% CI): 0.14 (0.03, 0.64), *p* = 0.01] lower for thiamin, 86% [OR (95% CI): 0.14 (0.03, 0.66), *p* = 0.01] lower for vitamin B6, and 87% [OR (95% CI): 0.13 (0.03, 0.59), *p* = 0.008] lower for pantothenic acid dietary intake compared with the lowest tertile.

Considering minerals, in the highest tertile, the odds of PD decreased more than 80% with dietary intake of magnesium [OR (95% CI): 0.11 (0.02, 0.50), *p* = 0.004], zinc [OR (95% CI): 0.14 (0.03, 0.69), *p* = 0.02], manganese [OR (95% CI): 0.15 (0.05, 0.49), *p* = 0.002], chromium [OR (95% CI): 0.20 (0.06, 0.61), *p* = 0.005], and phosphorus [OR (95% CI): 0.12 (0.03, 0.52), *p* = 0.005] compared with the lowest tertile.

### 3.3. Area under the Curve (AUC) of Vitamins and Minerals in Predicting Parkinson’s Disease

**Figure 1:**
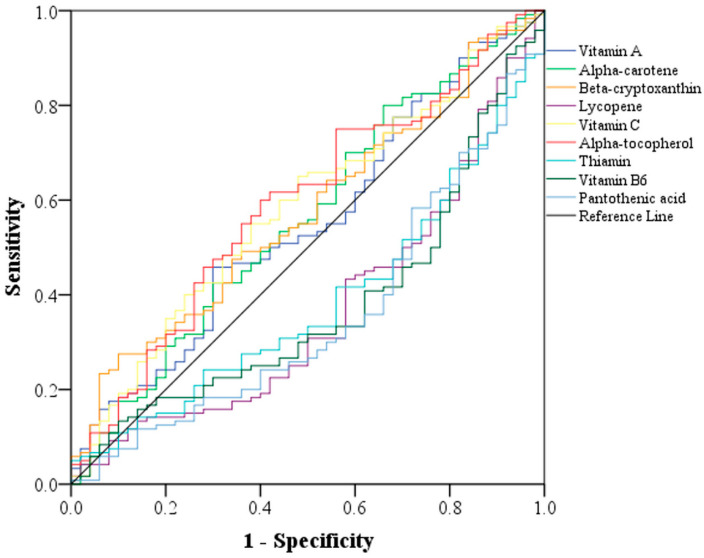
Receiver operating characteristic curve according to sensitivity (*y*-axis) and specificity (*x*-axis) to compare the predictive power of various vitamins concerning PD.

**Figure 2:**
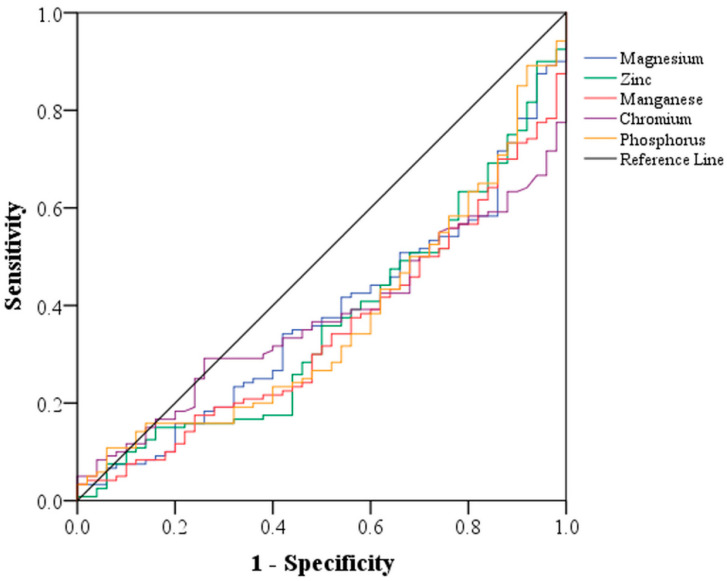
Receiver operating characteristic curve according to sensitivity (*y*-axis) and specificity (*x*-axis) to compare the predictive power of various minerals concerning PD.

According to ROC curve analysis, among the vitamins, dietary intake of lycopene, thiamin, vitamin B6, and pantothenic acid displayed clinical importance in predicting PD (Table 5). The area under the curve (AUC) of the four vitamins was approximately the same (Figure 1). However, the AUC was higher for vitamin A, α-carotene, β-cryptoxanthin, vitamin C, and α-tocopherol. Among the minerals, magnesium, zinc, manganese, chromium, and phosphorus exhibited clinical importance in predicting PD (Table 5). The AUC of the minerals did not considerably differ together (Figure 2).

### 3.4. Dietary Intake of Vitamins and Minerals and Severity of Parkinson’s Disease

As shown in Table 6, the total score of UPDRS was negatively correlated with dietary intake of β-carotene (β = −0.21, *p* = 0.049), vitamin C (β = −0.28, *p* = 0.01), riboflavin (β = −0.32, *p* = 0.05), vitamin B6 (β = −0.40, *p* = 0.03), biotin (β = −0.47, *p* < 0.001), and pantothenic acid (β = −0.51, *p* = 0.004). Non-motor aspects of experiences of daily living did not significantly correlate with vitamins, but motor aspects of experiences of daily living were negatively correlated with riboflavin intake (β = −0.35, *p* = 0.03). Motor examination was negatively correlated with β-carotene (β = −0.27, *p* = 0.01), lutein (β = −0.20, *p* = 0.048), vitamin C (β = −0.35, *p* = 0.001), vitamin B6 (β = −0.53, *p* = 0.004), biotin (β = −0.54, *p* < 0.001), and pantothenic acid (β = −0.60, *p* = 0.001). There was a significant correlation between dietary intake of riboflavin (β = −0.35, *p* = 0.02), vitamin B6 (β = −0.42, *p* = 0.01), vitamin B12 (β = −0.36, *p* = 0.002), and pantothenic acid (β = −0.36, *p* = 0.03) with motor complications.

As shown in Table 7, the total score of UPDRS was negatively correlated with dietary intake of phosphorus (β = −0.40, *p* = 0.02), magnesium (β = −0.45, *p* = 0.002), zinc (β = −0.38, *p* = 0.02), manganese (β = −0.21, *p* = 0.05), chromium (β = −0.22, *p* = 0.03), and strongly with potassium (β = −0.77, *p* < 0.001). Non-motor aspects of experiences of daily living were negatively correlated with dietary intake of fluorine (β = −0.18, *p* = 0.048) and potassium (β = −0.34, *p* = 0.06). Motor aspects of experiences of daily living were negatively correlated with dietary intake of calcium (β = −0.38, *p* = 0.004) and potassium (β = −0.39, *p* = 0.04). Motor examination symptoms were negatively correlated with dietary intake of phosphorus (β = −0.41, *p* = 0.02), magnesium (β = −0.52, *p* < 0.001), zinc (β = −0.48, *p* = 0.003), manganese (β = −0.23, *p* = 0.04), chromium (β = −0.26, *p* = 0.01), and potassium (β = −0.84, *p* < 0.001). There was a significant negative correlation between dietary intake of phosphorus (β = −0.43, *p* = 0.01), magnesium (β = −0.36, *p* = 0.01), zinc (β = −0.32, *p* = 0.04), manganese (β = −0.20, *p* = 0.05), chromium (β = −0.19, *p* = 0.04), and potassium (β = −0.66, *p* < 0.001) with motor complications.

## 4. Discussion

In the present study, dietary intake of vitamin E did not significantly differ between PD patients and controls. Dietary intake of vitamin E was not correlated with the odds and severity of PD. But patients with PD had a higher intake of α-tocopherol. High intake of α-tocopherol was correlated with increased odds of PD, and its predictive power concerning the risk of PD was stronger relative to other vitamins.

Previous studies have mostly focused on total vitamin E and suggested a protective effect for it against PD [19,20,21]. However, investigation regarding dietary intake of α-tocopherol in patients with PD is scarce. Ascherio et al., in a randomized controlled trial study on eight hundred individuals with early PD, showed that the risk of PD was reduced only among patients who had not been treated with α-tocopherol compared to those treated [22], which in a way may confirm the finding of the present study. Abbott et al., in a cohort of 8006 male individuals, did not find a clear relation between α-tocopherol and clinical PD. However, in this study, the method of dietary intake measure has not been presented [23].

Kim et al. found no significant difference in serum levels of α-tocopherol between PD patients and controls [24]. Also, Férnandez-Calle et al. [25] and Nicoletti et al. [26] in two separate studies reported that the circulating levels of α-tocopherol (vitamin E) did not significantly differ between the PD patients and normal individuals. The dietary intake assessment method, disease duration, and stage of the disease are factors that may be responsible for inconsistent findings across the studies.

In the current study, patients with PD had a lower intake of pantothenic acid, and a high intake of pantothenic acid was correlated with reduced odds of PD. Dietary intake of pantothenic acid was negatively correlated with PD severity and symptoms of motor examination and complication.

Reduced levels of pantothenic acid have been reported in several brain regions by previous investigations. Scholefield et al. compared nine post-mortem brain arias of patients with PD dementia and controls and found significantly reduced pantothenic acid levels in the cerebellum, substantia nigra, and medulla of PD cases [27]. Decreased levels of pantothenic acid have also been reported in brain structures of other neurodegenerative diseases such as Alzheimer’s disease [28]. However, Abbott et al., in a cohort of 8006 male individuals, did not find a clear relationship between dietary pantothenic acid intake and clinical PD [23].

Shao et al., in metabolic profiling of PD and in pathway analysis, found that metabolic impairment in pantothenate biosynthesis might have contributed to the pathogenesis of PD [29]. Vascellari et al., in a study on 64 patients with PD, reported that alterations in gut microbiota and metabolome were significantly correlated with the reduction of several fecal metabolites including pantothenic acid [30]. Baldini et al. showed that the gut microbial pantothenic acid production potential was positively associated with a greater non-motor symptom score, both in patients with PD and in controls [31]. All the studies indicate the involvement of pantothenic acid in the pathogenesis of PD.

Semenovich et al., in an animal model of PD, showed that pantothenic acid derivatives including panthenol and pantethine modulated oxidative stress parameters and thiol–disulfide balance in the brain structures [32]. This may point out that pantothenic acid possibly exerts its protective role against PD by reducing oxidative stress.

In the present study, PD’s severity and motor symptoms were also negatively correlated with β-carotene, vitamin C, riboflavin, vitamin B6, and biotin intake. Findings regarding the association of carotenoids and B vitamins with the development of PD remain controversial. In agreement with our findings, Kim et al., in a study on 104 patients with PD, reported that α- and β-carotenes were adversely associated with the UPDRS motor score [24]. Jamali et al., in a study on rats, showed that β-carotene has a therapeutic effect on PD conditions and inhibits the substantia nigra dopaminergic cell death [33]. In addition, Wu et al., in a meta-analysis study, concluded that consumption of dietary β-carotene, but not vitamin A intake, may have a protective effect against PD [34]. However, Hughes et al., in a prospective cohort study of 1036 people with PD, reported no significant association between dietary intakes of carotenoids and the risk of PD [35].

Scientific investigations have revealed the importance of the vitamin B family in managing neurological diseases like Parkinson’s. Several studies have documented that vitamin B6 has been associated with a lower risk of developing PD. Murakami et al. showed that a low intake of vitamin B6, but not riboflavin, was independently correlated with an augmented risk of PD [36]. In addition, Shen, in a meta-analysis study, found a preventive effect for dietary intake of vitamin B6, but not folate and vitamin B12, against PD [37]. However, the evidence is still limited, and further research is needed to explore the potential therapeutic applications of the vitamin B family, in particular concerning biotin, regarding PD.

Multiple studies have demonstrated a significant relationship between low levels of serum thiamine and the risk of PD [38,39,40] and reported that thiamine supplements may have favorable clinical effects against PD [41,42]. It has been shown that thiamine deficiency can reduce the dopamine level in the animal’s striatum [43], and intrastriatal administration of thiamin induces dopamine release [44]. Therefore, it appears that thiamine might be contributed to the pathogenesis of PD through involvement in dopamine synthesis.

Lycopene is an aliphatic hydrocarbon carotenoid with potent anti-inflammatory, anti-oxidative, and anti-proliferative properties [45,46]. According to evidence, lycopene has prophylactic and therapeutic effects in various neurodegenerative disorders including PD [47]. Prema et al. found that lycopene administration protected 1-methyl-4-phenyl-1,2,3,6-tetrahydropyridine-induced depletion of striatal dopamine in PD model mice, indicating that lycopene treatment may protect dopaminergic neurons against PD-inducing stimuli [48]. Some other mechanisms for the neuroprotective effects of lycopene include inhibition of neuronal apoptosis, prevention of oxidative stress and neuroinflammation, and promotion of mitochondrial function [47].

Vitamin C is an important neuromodulator in dopaminergic neurons and its deficiency is known to influence brain performance and is associated with parkinsonism. Liu et al. demonstrated a causal association between genetically increased plasma vitamin C levels and decreased PD age at onset in individuals of European descent [49]. Hantikainen et al., in a cohort study on 43,865 participants with 17.6 years of follow-up time, reported that dietary vitamin C consumption might be negatively associated with the risk of PD [19]. Hughes et al. showed that intake of dietary vitamin C significantly lowered the risk of PD, but the 4-year-lag analysis did not support the finding [35]. Lowered lymphocytes vitamin C level has also been observed in patients with severe PD [50]. The protective effect of vitamin C against developing PD might be due to improving levodopa absorption, increasing the production of dihydroxyphenylalanine, contributing to the differentiation of dopaminergic cells, and reducing levodopa and the MPTP toxicity [51].

In the present study, dietary intake of several minerals including magnesium, zinc, manganese, selenium, chromium, and phosphorus were lower in patients with PD. High magnesium, zinc, manganese, chromium, and phosphorus intake correlated with reduced PD odds. The UPDRS total score and motor symptoms in PD patients were negatively correlated with phosphorus, magnesium, zinc, manganese, chromium, and potassium intake. Palavra et al., in a study of 103 patients with PD and 81 healthy individuals, found no significant difference between the two groups regarding the dietary intake of minerals including iron, calcium, magnesium, potassium, and zinc [52]. But the intake of manganese, potassium, and zinc was lower in patients when expressed per 1000 kJ intake [52]. In patients with PD, reduced potassium, zinc, and manganese levels were found in the hippocampus, middle temporal gyrus, and motor cortex; reduced manganese was also found in substantia nigra and medulla oblongata, and lower selenium and magnesium were found in the motor cortex [53]. Talebi et al., in a meta-analysis study, found that the risk of PD was reduced for each unit (1 mg/d) increment in dietary zinc intake [54]. Adani et al., in a meta-analysis study, showed lower levels of zinc both in cerebrospinal fluid and serum of patients with PD compared with controls [55]. According to the evidence, the metals may impact levodopa therapy in PD patients [56] and thus on symptoms of the disease.

Magnesium is vital for various cell actions such as the transport of calcium and potassium ions, proliferation of cells, and regulating nerve signal transmission. Experimental evidence showed that low magnesium intake over two generations led to an intense loss of dopaminergic neurons exclusively in the substantia nigra, and magnesium treatment inhibited neurite and neuron pathology in a rat PD model [57]. It seems that magnesium protects dopaminergic neurons from degeneration. Moreover, magnesium may have a protective effect against PD by taming neuroinflammation [58].

Zinc ions increase the capacity of human serum albumin, a plentiful transport protein in the blood, to better inhibit aggregation of α-synuclein, a protein that is directly connected to PD [59]. Dietary chromium has a trivalent state which is the biologically active form and is a component of metalloenzymes. It participates in the metabolism of macronutrients and oxidative state. Reduced circulating chromium is associated with hyperglycemia and insulin resistance [60]. Insulin resistance increases the development and progression of PD via abnormal expression of α-synuclein, enhanced production of reactive oxygen species, mitochondrial dysfunction, and deregulation of the Polo-Like Kinase 2 Signaling [61].

According to evidence, high cortisol levels dysregulate the hypothalamic–pituitary–adrenal (HPA) axis which may promote symptoms of PD [62]. On the other hand, low levels of serum phosphorus increase the risk of PD [63]. Håglin et al. found a negative association between phosphate and cortisol in patients with PD and concluded that low phosphate levels may affect cognition and motor function in PD [64]. Taken together, it seems low phosphate increases cortisol levels, which in turn causes dysregulation of the HPA axis, ultimately increasing the risk of PD.

Interestingly, we observed a strong inverse association between potassium intake and disease severity and symptoms. It has been reported that the deficiency of potassium ions in the nervous system leads to dysfunction of certain nerve cells or brain regions and then to neurological and mental diseases [65]. Cisternas et al. showed that the increase in potassium intake causes a recovery in the generation of neuronal plasticity, a decrease in the expression of histopathological markers, and improves cognitive performance in a model of Alzheimer’s disease [66]. Furthermore, recently, the role of potassium channels in the progression or treatment of PD has attracted the attention of researchers and has been known as a potential therapeutic target for PD [67,68]. It appears that blockade or activation of these channels possibly led to the regulation of nerve cells’ potassium which subsequently leads to a reduction of the clinical complications of the disease. However, more studies are needed to confirm the issue.

### Limitations of the Study

Dietary assessment using FFQ is generally based on foods consumed in the previous year, therefore participants may forget the type, amount, and frequency of a certain food consumed. This may influence the estimation of vitamins and minerals intake. Given the case-control design of the study, causality cannot be inferred based on our findings.

## 5. Conclusions

High vitamin A, α-carotene, β-cryptoxanthin, vitamin C, and α-tocopherol and low lycopene, thiamin, vitamin B6, pantothenic acid, magnesium, zinc, manganese, chromium, and phosphorous intake were correlated with increased odds of PD. High pantothenic acid, β-carotene, vitamin C, riboflavin, vitamin B6, biotin, phosphorus, magnesium, zinc, manganese, chromium, and potassium intake were correlated with reduced disease severity and motor dysfunction. The findings indicate that adequate dietary intake of vitamins and minerals may have a preventive effect on developing PD and progression of motor decline. Prospective cohort studies are required to investigate the long-term effects of vitamins and minerals on the incidence of PD, randomized controlled trials are needed to establish causal conclusions, and experimental studies are needed to explore the potential pathways and mechanisms of action.

## Figures and Tables

**Table 1:** Demographic characteristics of participants in patient and healthy groups.

Variable	Participants		*p*-Value
	Patients (*n* = 120)	Healthy (*n* = 50)	
Age *	60.8 ± 9.8	60.4 ± 9.8	0.93
Gender ^†^			0.98
Men	79 (65.8)	33 (66)	
Women	41 (34.2)	17 (34)	
Body Mass Index *	25.3 ± 4.3	26.0 ± 5.0	0.34
Comorbidities ^†^			
Diabetes mellitus	10 (8.3)	-	
Hypertension	29 (24.2)	-	
Thyroid disorders	14 (11.7)	-	
Cardiovascular disease	15 (12.5)	-	
Smoking ^†^ (%)	11 (9.2)	12 (24.0)	0.01
Total UPDRS *	46.2 ± 25.2	-	
Symptoms of non-motor aspects of experiences of daily living *	7 ± 5.4	-	
Symptoms of motor aspects of experiences of daily living *	12 ± 7.2	-	
Symptoms of motor examination *	26 ± 15.6	-	

* Data are presented as mean ± SD. ^†^ Data are presented as frequency (percent).

**Table 2:** Comparison of dietary intake of vitamins between groups.

	Parkinson’s Disease (*n* = 120)	Healthy Individuals (*n* = 50)	*p*
Vitamin A (mg) *	424.99 ± 173.87	391.64 ± 155.69	0.24
β-Carotene (mg) ^†^	2260.01 (1220.86)	2125.50 (1613.05)	0.48
α-Carotene (mg) ^†^	360.02 (320.82)	242.01 (242.01)	0.21
Lutein (mg) ^†^	1058.08 (602.21)	1234.79 (621.89)	0.44
β-Cryptoxanthin (mg) ^†^	127.71 (129.45)	116.53 (87.74)	0.14
Lycopene (mg) *	4113.21 ± 1749.60	4760.59 ± 1638.56	0.03
Vitamin C (mg) *	92.13 ± 41.84	80.92 ± 38.86	0.11
Vitamin D (µg) ^†^	0.95 (1.20)	0.87 (1.08)	0.92
Vitamin E (mg) *	11.60 ± 3.22	11.08 ± 2.92	0.33
α-Tocopherol (mg) *	7.26 ± 2.10	6.59 ± 1.83	0.05
Thiamin (mg) *	1.72 ± 0.49	1.89 ± 0.39	0.03
Riboflavin (mg) *	1.58 ± 0.52	1.73 ± 0.48	0.10
Niacin (mg) *	19.34 ± 5.39	19.84 ± 4.50	0.56
Vitamin B6 (mg) *	1.53 ± 0.40	1.69 ± 0.35	0.02
Folate (mg) *	425.47 ± 107.25	456.79 ± 95.02	0.07
Vitamin B_12_ (mg) ^†^	2.70 (2.09)	3.56 (2.27)	0.03
Biotin *	28.29 ± 9.56	31.27 ± 8.41	0.06
Pantothenic acid (mg) *	4.55 ± 1.30	5.18 ± 1.23	0.004
Vitamin K (mg) ^†^	133.50 (76.58)	139.63 (85.42)	0.30

* Data are presented as mean ± SD.^†^ Data are presented as median (interquartile range).

**Table 3:** Comparison of dietary intake of minerals between groups.

	Parkinson’s Disease (*n* = 120)	Healthy Individuals (*n* = 50)	*p*
Iron (mg) *	14.42 ± 3.84	15.20 ± 3.52	0.22
Magnesium (mg) *	360.68 ± 106.95	400.80 ± 90.50	0.02
Zinc (mg) *	10.09 ± 3.14	11.30 ± 2.73	0.02
Copper (mg) *	1.45 ± 0.39	1.55 ± 0.37	0.14
Manganese (mg) *	6.97 ± 2.66	8.19 ± 2.17	0.005
Selenium (mg) *	114.01 ± 40.88	129.27 ± 33.63	0.02
Chromium *	0.16 ± 0.12	0.20 ± 0.07	0.01
Potassium (mg) *	2754.88 ± 812.78	2935.23 ± 817.90	0.19
Calcium (mg) *	791.62 ± 353.43	888.02 ± 330.11	0.10
Phosphorus (mg) *	1248.62 ± 402.42	1403.77 ± 343.77	0.02
Fluorine ^†^	1925.65 (1660.77)	2624.42 (1912.97)	0.25

* Data are presented as mean ± SD. ^†^ Data are presented as median (interquartile range).

**Table 4:** Correlation between dietary intake of vitamins and minerals with odds of Parkinson’s disease.

	Q2 (*n* = 57)	*p*	Q3 (*n* = 57)	*p*
Vitamin A (mg)	1.52 (0.59, 3.89)	0.38	3.57 (1.05, 12.10)	0.04
β-Carotene (mg)	1.59 (0.64, 3.93)	0.32	1.66 (0.61, 4.56)	0.32
α-Carotene (mg)	1.92 (0.76, 4.84)	0.16	3.27 (1.18, 9.04)	0.02
Lutein (Mg)	1.57 (0.59, 4.16)	0.36	0.96 (0.35, 2.60)	0.94
β-Cryptoxanthin (mg)	2.10 (0.85, 5.21)	0.11	3.41 (1.21, 9.60)	0.02
Lycopene (mg)	0.53 (0.20, 1.46)	0.22	0.24 (0.09, 0.67)	0.006
Vitamin C (mg)	1.21 (0.51, 2.89)	0.66	3.11 (1.11, 8.69)	0.03
Vitamin D (ug)	0.87 (0.36, 2.08)	0.75	1.15 (0.46, 2.86)	0.77
Vitamin E (mg)	1.49 (0.62, 3.60)	0.37	1.92 (0.69, 5.36)	0.21
α-Tocopherol (mg)	2.72 (1.09, 6.78)	0.03	4.31 (1.51, 12.28)	0.006
Thiamin (mg)	0.29 (0.09, 0.94)	0.04	0.14 (0.03, 0.64)	0.01
Riboflavin (mg)	0.39 (0.14, 1.10)	0.08	0.27 (0.07, 1.09)	0.07
Niacin (mg)	1.23 (0.44, 3.43)	0.70	0.75 (0.22, 2.52)	0.64
Vitamin B6 (mg)	0.38 (0.13, 1.10)	0.07	0.14 (0.03, 0.66)	0.01
Folate (Mg)	0.70 (0.23, 2.11)	0.52	0.28 (0.07, 1.14)	0.07
Vitamin B12 (mg)	0.80 (0.29, 2.19)	0.66	0.33 (0.11, 1.03)	0.06
Biotin	0.53 (0.21, 1.38)	0.19	0.65 (0.21, 1.99)	0.45
Pantothenic acid (mg)	0.47 (0.15, 1.45)	0.19	0.13 (0.03, 0.59)	0.008
Vitamin K (mg)	1.18 (0.46, 3.04)	0.73	0.66 (0.24, 1.77)	0.41
Iron (mg)	1.14 (0.41, 3.18)	0.80	0.46 (0.14, 1.54)	0.21
Magnesium (mg)	0.14 (0.04, 0.46)	0.001	0.11 (0.02, 0.50)	0.004
Zinc (mg)	0.40 (0.12, 1.29)	0.12	0.14 (0.03, 0.69)	0.02
Copper (mg)	1.13 (0.41, 3.08)	0.82	0.55 (0.17, 1.78)	0.32
Manganese (mg)	0.29 (0.10, 0.85)	0.02	0.15 (0.05, 0.49)	0.002
Selenium (mg)	0.40 (0.14, 1.12)	0.08	0.35 (0.11, 1.06)	0.06
Chromium	0.15 (0.05, 0.45)	0.001	0.20 (0.06, 0.61)	0.005
Potassium (mg)	0.59 (0.21, 1.69)	0.33	0.23 (0.05, 1.05)	0.06
Calcium (mg)	0.68 (0.25, 1.83)	0.44	0.37 (0.11, 1.26)	0.11
Phosphorus (mg)	0.40 (0.13, 1.22)	0.11	0.12 (0.03, 0.52)	0.005
Fluorine	1.14 (0.47, 2.77)	0.77	0.92 (0.39, 2.20)	0.86

Tertile 1 (*n* = 56) was used as reference. Age, gender, smoking, energy intake, body mass index, diseases (diabetes, hypertension, cardiovascular diseases, mental, neurologic, and thyroid diseases) were considered as confounders in analysis.

**Table 5:** Area under the curve (AUC) of vitamins and minerals in predicting Parkinson’s disease.

Vitamins	AUC (95% CI)	*p*	Minerals	AUC (95% CI)	*p*
Vitamin A	0.55 (0.45, 0.64)	0.31	Magnesium	0.38 (0.29, 0.46)	0.01
α-Carotene	0.56 (0.46, 0.66)	0.21	Zinc	0.37 (0.28, 0.45)	0.006
β-Cryptoxanthin	0.57 (0.48, 0.66)	0.14	Manganese	0.35 (0.26, 0.43)	0.002
Lycopene	0.37 (0.28, 0.46)	0.007	Chromium	0.37 (0.29, 0.46)	0.009
Vitamin C	0.58 (0.49, 0.68)	0.09	Phosphorus	0.37 (0.28, 0.46)	0.007
α-Tocopherol	0.59 (0.50, 0.68)	0.06			
Thiamin	0.38 (0.30, 0.47)	0.02			
Vitamin B6	0.37 (0.28, 0.46)	0.01			
Pantothenic acid	0.35 (0.26, 0.44)	0.003			

**Table 6:** Correlation between dietary intake of vitamins and severity of Parkinson’s disease.

	Total Score of UPDRS		Non-Motor Aspects of Experiences of Daily Living		Motor Aspects of Experiences of Daily Living		Motor Examination		Motor Complications	
	β (95% CI)	*p*	β (95% CI)	*p*	β (95% CI)	*p*	β (95% CI)	*p*	β (95% CI)	*p*
Vitamin A (RAE)(Mg)	−0.12 (−0.05, 0.02)	0.31	−0.07 (−0.01, 0.005)	0.53	−0.050 (−0.012, 0.008)	0.68	−0.14 (−0.03, 0.01)	0.25	−0.07 (−0.005, 0.002)	0.52
β-Carotene (Mg)	−0.21 (−0.01, 0.00)	0.049	−0.08 (−0.001, 0.001)	0.41	−0.06 (−0.002, 0.001)	0.55	−0.27 (−0.01,−0.001)	0.01	−0.09 (−0.001, 0.00)	0.37
α-Carotene (mg)	−0.10 (−0.03, 0.01)	0.32	0.002 (−0.004, 0.004)	0.98	0.02 (−0.005, 0.01)	0.87	−0.15 (−0.02, 0.003)	0.13	−0.12 (−0.003, 0.001)	0.21
Lutein (mg)	−0.15 (−0.01, 0.002)	0.14	−0.09 (−0.002, 0.001)	0.36	−0.02 (−.0003, 0.002)	0.85	−0.20 (−0.01,−0.00004)	0.048	−0.03 (−0.001, 0.001)	0.73
β-Cryptoxanthin (mg)	−0.08 (−0.08, 0.03)	0.45	0.14 (−0.003, 0.02)	0.14	0.04 (−0.01, 0.02)	0.71	−0.18 (−0.06, 0.004)	0.09	−0.11 (−0.01, 0.002)	0.24
Lycopene (mg)	−0.12 (−0.005, 0.001)	0.23	−0.05 (−0.001, 0.0004)	0.63	0.003 (−0.001, 0.001)	0.97	−0.16 (−0.003, 0.0003)	0.12	−0.14 (0.00, 0.00)	0.14
Vitamin C (mg)	−0.28 (−0.29,−0.04)	0.01	−0.08 (−0.04, 0.01)	0.41	−0.11 (−0.05, 0.02)	0.31	−0.35 (−0.20,−0.05)	0.001	−0.17 (−0.02, 0.002)	0.08
Vitamin D (µg)	−0.17 (−11.49, 0.98)	0.10	−0.14 (−2.15, 0.37)	0.17	−0.11 (−2.68, 0.82)	0.29	−0.16 (−6.83, 0.87)	0.13	−0.14 (−1.06, 0.15)	0.14
Vitamin E (mg)	−0.09 (−2.43, 1.02)	0.42	−0.05 (−0.43, 0.26)	0.62	−0.11 (−0.73, 0.22)	0.30	−0.08 (−1.44, 0.69)	0.49	0.02 (−0.15, 0.18)	0.88
α-Tocopherol (mg)	−0.08 (−3.57, 1.73)	0.49	−0.05 (−0.66, 0.41)	0.65	−0.10 (−1.08, 0.40)	0.36	−0.07 (−2.14, 1.13)	0.54	0.04 (−0.21, 0.31)	0.72
Thiamin (mg)	0.003 (−17.07, 17.37)	0.99	−0.06 (−4.12, 2.81)	0.71	−0.04 (−5.44, 4.14)	0.79	0.05 (−9.08, 12.13)	0.78	−0.01 (−1.75, 1.60)	0.93
Riboflavin (mg)	−0.32 (−30.86, 0.33)	0.05	−0.20 (−5.21, 1.14)	0.21	−0.35 (−9.19,−0.56)	0.03	−0.22 (−16.28, 3.11)	0.18	−0.35 (−3.27,−0.26)	0.02
Niacin (mg)	0.09 (−0.86, 1.72)	0.51	0.06 (−0.20, 0.32)	0.63	0.24 (−0.04, 0.67)	0.08	0.01 (−0.75, 0.84)	0.92	0.01 (−0.12, 0.13)	0.92
Vitamin B6 (mg)	−0.40 (−47.26,−2.05)	0.03	0.02 (−4.41, 4.90)	0.92	−0.10 (−8.26, 4.57)	0.57	−0.53 (−34.02,−6.66)	0.004	−0.42 (−4.90,−0.53)	0.01
Folate (mg)	0.19 (−0.03, 0.12)	0.24	−0.03 (−0.02, 0.01)	0.82	0.12 (−0.01, 0.03)	0.43	0.23 (−0.01, 0.08)	0.15	0.16 (−0.003, 0.01)	0.30
Vitamin B12 (mg)	0.08 (−2.52, 5.00)	0.52	0.07 (−0.54, 0.98)	0.57	0.03 (−0.94, 1.16)	0.83	0.10 (−1.38, 3.24)	0.43	−0.02 (−0.39, 0.34)	0.88
Biotin	−0.47 (−1.89,−0.61)	<0.001	−0.20 (−0.25, 0.02)	0.09	−0.20 (−0.34, 0.04)	0.12	−0.54 (−1.27,−0.50)	<0.001	−0.36 (−0.16,−0.04)	0.002
Pantothenic acid (mg)	−0.51 (−16.66,−3.23)	0.004	−0.14 (−1.99, 0.81)	0.41	−0.26 (−3.36, 0.49)	0.14	−0.60 (−11.27,−3.12)	0.001	−0.36 (−1.39,−0.06)	0.03
Vitamin K (mg)	−0.16 (−0.11, 0.01)	0.10	−0.08 (−0.02, 0.01)	0.39	−0.08 (−0.02, 0.01)	0.40	−0.19 (−0.07, 0.001)	0.06	−0.05 (−0.01, 0.004)	0.59

Age, gender, smoking, energy intake, body mass index, diabetes, hypertension, cardiovascular diseases, mental, neurologic, and thyroid diseases were considered as confounders in the analysis.

**Table 7:** Correlation between dietary intake of minerals and severity of Parkinson’s disease.

	Total Score of UPDRS		Non-Motor Aspects of Experiences of Daily Living		Motor Aspects of Experiences of Daily Living		Motor Examination		Motor Complications	
	β (95% CI)	*p*	β (95% CI)	*p*	β (95% CI)	*p*	β (95% CI)	*p*	β (95% CI)	*p*
Calcium (mg)	−0.25 (−0.04, 0.001)	0.07	−0.09 (−0.005, 0.002)	0.46	−0.38 (−0.01, −0.003)	0.004	−0.15 (−0.02, 0.005)	0.26	−0.24 (−0.004, 0.00)	0.06
Iron (mg)	−0.06 (−2.38, 1.58)	0.69	−0.10 (−0.55, 0.25)	0.46	0.11 (−0.34, 0.75)	0.46	−0.10 (−1.63,0.81)	0.50	−0.07 (−0.24, 0.14)	0.62
Phosphorus (mg)	−0.40 (−0.05, −0.004)	0.02	−0.08 (−0.005, 0.003)	0.63	−0.30 (−0.01, 0.001)	0.07	−0.41 (−0.03, −0.003)	0.02	−0.43 (−0.005, −0.001)	0.01
Magnesium (mg)	−0.45 (−0.17, −0.04)	0.002	−0.24 (−0.03, 0.001)	0.08	−0.14 (−0.03, 0.01)	0.32	−0.52 (−0.12, −0.04)	<0.001	−0.36 (−0.01, −0.002)	0.01
Zinc (mg)	−0.38 (−5.62, −0.45)	0.02	−0.10 (−0.71, 0.36)	0.51	−0.08 (−0.93, 0.54)	0.60	−0.48 (−3.97, −0.83)	0.003	−0.32 (−0.52, −0.01)	0.04
Copper (mg)	−0.05 (−22.57, 15.60)	0.72	−0.05 (−4.54, 3.15)	0.72	0.15 (−2.52, 8.06)	0.30	−0.13 (−16.92, 6.52)	0.38	−0.05 (−2.21, 1.50)	0.71
Manganese (mg)	−0.21 (−4.05, 0.03)	0.05	−0.18 (−0.79, 0.04)	0.07	−0.04 (−0.68, 0.47)	0.72	−0.23 (−2.59, −0.08)	0.04	−0.20 (−0.39, 0.003)	0.05
Selenium (mg)	−0.16 (−0.24, 0.05)	0.20	−0.13 (−0.05, 0.01)	0.25	0.05 (−0.03, 0.05)	0.67	−0.20 (−0.17, 0.01)	0.09	−0.16 (−0.02, 0.004)	0.14
Fluorine	−0.05 (−0.005, 0.003)	0.59	−0.18 (−0.001, 0.000)	0.048	−0.03 (−0.001, 0.001)	0.78	0.01 (−0.002, 0.002)	0.95	−0.11 (−0.001, 0.00)	0.26
Chromium	−0.22 (−91.70, −5.61)	0.03	−0.13 (−14.93, 2.67)	0.17	−0.05 (−15.67, 8.82)	0.58	−0.26 (−61.06, −8.46)	0.01	−0.19 (−8.54, −0.14)	0.04
Potassium (mg)	−0.773 (−0.035, −0.013)	<0.001	−0.34 (−0.005, 0.000)	0.06	−0.39 (−0.01, −0.0002)	0.04	−0.84 (−0.02, −0.01)	<0.001	−0.66 (−0.003, −0.001)	<0.001

Age, gender, smoking, energy intake, body mass index, diabetes, hypertension, cardiovascular diseases, mental, neurologic, and thyroid were considered as confounders in the analysis.

## Data Availability

The data presented in this study are available on request from the corresponding author.

## Supplementary Materials

The following supporting information can be downloaded at: https://www.mdpi.com/article/10.3390/brainsci13071119/s1, Supplementary Table S1. Comparison of dietary intake of vitamins between females and males; Supplementary Table S2. Comparison of dietary intake of minerals between females and males
