# Supplementary material for: What Dietary Vitamins and Minerals Might Be Protective against Parkinson’s Disease?
Source: Brain Sci. 2023 Jul 24;13(7):1119. doi: 10.3390/brainsci13071119 (PMC10377174; doi:10.3390/brainsci13071119)
Supplement: Supplementary file 1 [file brainsci-13-01119-s001.zip › Supplementary Table S1.pdf]

Supplementary Table S1. Comparison of dietary intake of vitamins between females and males

	Total			Parkinson's disease			Healthy individuals		
	**Female** (n=58)	**Male** (n=112)	**p**	**Female** (n=41)	**Male** (n=79)	**p**	**Female** (n=17)	**Male** (n=33)	**p**
**Vitamin A (mg)**	453.02 ± 196.53	395.59 ± 149.97	**0.05**	448.58 ± 203.09	412.75 ± 156.60	0.33	463.71 ± 185.23	354.51 ± 125.61	**0.04**
**β-Carotene (mg)**	2766.05 ± 1339.90	1339.90 ± 1096.18	**0.03**	2764.93 ± 1401.06	2394.62 ± 1163.16	0.17†	2768.76 ± 1220.02	2111.40 ± 901.08	**0.04**
**α-Carotene (mg)**	398.17 ± 307.50	320.27 ± 263.83	0.09†	413.16 ± 321.04	332.44 ± 256.38	0.18†	362.01 ± 277.89	291.14 ± 282.81	0.33†
**Lutein (mg)**	1323.34 ± 719.40	1173.12 ± 503.98	0.23†	1334.64 ± 778.18	1159.78 ± 525.91	0.31†	1296.10 ± 573.35	1205.05 ± 453.20	0.54
**β-Cryptoxanthin (mg)**	155.65 ± 87.51	140.15 ± 89.24	0.19†	157.31 ± 94.32	151.88 ± 95.38	0.76†	151.66 ± 70.82	112.07 ± 65.57	**0.048**†
**Lycopene (mg)**	4430.81 ± 1624.67	4237.74 ± 1797.76	0.49	4277.08 ± 1655.99	4028.16 ± 1800.67	0.46	4801.59 ± 1530.02	4739.47 ± 1714.46	0.90
**Vitamin C (mg)**	98.32 ± 47.51	83.92 ± 36.78	**0.047**	96.79 ± 46.11	89.71 ± 39.54	0.38	102.01 ± 51.99	70.05 ± 24.54	**0.03**†
**Vitamin D (µg)**	1.19 ± 0.85	1.18 ± 0.89	0.98†	1.18 ± 0.87	1.17 ± 0.81	0.82†	1.22 ± .84	1.21 ± 1.08	0.67†
**Vitamin E (mg)**	11.53 ± 3.93	11.41 ± 2.64	0.83	11.99 ± 4.00	11.40 ± 2.73	0.34	10.41 ± 3.64	11.43 ± 2.46	0.24
**α-Tocopherol (mg)**	7.34 ± 2.57	6.91 ± 1.70	0.25	7.67 ± 2.59	7.04 ± 1.78	0.12	6.56 ± 2.43	6.61 ± 1.48	0.94
**Thiamin (mg)**	1.56 ± 0.44	1.87 ± 0.45	**<0.001**	1.51 ± 0.46	1.82 ± 0.47	**0.001**	1.68 ± 0.36	2.00 ± 0.36	**0.004**
**Riboflavin (mg)**	1.62 ± 0.62	1.63 ± 0.45	0.92	1.53 ± 0.61	1.61 ± 0.47	0.42	1.83 ± 0.60	1.67 ± 0.41	0.33
**Niacin (mg)**	17.14 ± 4.39	20.70 ± 5.09	**<0.001**	17.14 ± 4.75	20.48 ± 5.37	**0.001**	17.14 ± 3.50	21.24 ± 4.35	**0.002**
**Vitamin B6 (mg)**	1.52 ± 0.43	1.61 ± 0.38	0.13	1.45 ± 0.42	1.58 ± 0.39	0.10	1.68 ± 0.42	1.70 ± 0.32	0.86
**Folate (mg)**	392.63 ± 93.76	456.46 ± 103.50	**<0.001**	390.57 ± 96.92	443.59 ± 108.44	**0.01**	397.58 ± 88.30	487.29 ± 84.29	**0.001**
**Vitamin B12 (mg)**	3.12 ± 1.60	3.22 ± 1.56	0.56†	2.95 ± 1.62	3.12 ± 1.65	0.44†	3.52 ± 1.51	3.44 ± 1.32	0.85
**Biotin**	27.25 ± 9.91	30.16 ± 8.86	**0.05**	25.86 ± 9.91	29.55 ± 9.18	**0.04**	30.60 ± 9.37	31.61 ± 8.00	0.69
**Pantothenic acid (mg)**	4.50 ± 1.40	4.86 ± 1.24	0.09	4.27 ± 1.35	4.70 ± 1.26	0.08	5.06 ± 1.41	5.24 ± 1.15	0.64
**Vitamin K (Mg)**	150.41 ± 83.23	129.33 ± 76.49	0.11†	150.06 ± 84.27	129.10 ± 86.58	0.15†	151.26 ± 83.20	129.88 ± 44.94	0.48†

Data presented as mean ± SD. † Mann-Whitney test was used for analysis.
