# Supplementary material for: What Dietary Vitamins and Minerals Might Be Protective against Parkinson’s Disease?
Source: Brain Sci. 2023 Jul 24;13(7):1119. doi: 10.3390/brainsci13071119 (PMC10377174; doi:10.3390/brainsci13071119)
Supplement: Supplementary file 1 [file brainsci-13-01119-s001.zip › Supplementary Table S2.pdf]

Supplementary Table S2. Comparison of dietary intake of minerals between females and males

	**Total**			**Parkinson's disease**			**Healthy individuals**		
	**Female** (n=58)	**Male** (n=112)	**p**	**Female** (n=41)	**Male** (n=79)	**p**	**Female** (n=17)	**Male** (n=33)	**p**
**Calcium (mg)**	867.36 ± 426.71	795.44 ± 299.49	0.25	794.35 ± 411.60	790.20 ± 322.01	0.79†	1043.43 ± 422.54	807.96 ± 241.15	**0.04**
**Iron (mg)**	12.91 ± 3.45	15.55 ± 3.59	**<0.001**	12.86 ± 3.57	15.23 ± 3.74	**0.001**	13.02 ± 3.25	16.32 ± 3.14	**0.001**
**Phosphorus (mg)**	1231.83 ± 440.52	1326.57 ± 361.53	0.16	1151.84 ± 426.89	1298.84 ± 382.28	0.06	1424.75 ± 424.01	1392.96 ± 301.19	0.78
**Magnesium (mg)**	341.51 ± 108.97	388.52 ± 97.63	**0.005**	325.78 ± 107.41	378.79 ± 102.78	**0.009**	379.44 ± 106.28	411.81 ± 80.78	0.23
**Zinc (mg)**	9.64 ± 3.16	10.86 ± 2.94	**0.01**	9.11 ± 2.94	10.60 ± 3.14	**0.01**	10.92 ± 3.39	11.49 ± 2.35	0.49
**Copper (mg)**	1.33 ± 0.38	1.56 ± 0.36	**<0.001**	1.32 ± 0.37	1.52 ± 0.38	**0.005**	1.37 ± 0.41	1.64 ± 0.31	**0.01**
**Manganese (mg)**	5.89 ± 2.01	8.08 ± 2.53	**<0.001**	5.76 ± 2.27	7.60 ± 2.64	**<0.001**	6.18 ± 1.18	9.22 ± 1.82	**<0.001**
**Selenium (mg)**	97.33 ± 31.59	129.46 ± 38.69	**<0.001**	95.11 ± 35.43	123.82 ± 40.25	**<0.001**	102.71 ± 19.29	142.96 ± 31.26	**<0.001**
**Fluorine**	1954.92 ± 1056.22	2592.05 ± 1441.96	**0.01**†	2064.23 ± 1109.95	2392.31 ± 1460.78	0.37†	1691.29 ± 888.78	3070.22 ± 1294.91	**0.001**†
**Chromium**	0.13 ± 0.08	0.20 ± 0.11	**<0.001**†	0.12 ± 0.10	0.19 ± 0.12	**0.002**†	0.15 ± 0.04	0.23 ± 0.07	**<0.001**
**Potassium (mg)**	2851.70 ± 981.50	2785.25 ± 719.49	0.62	2734.15 ± 939.61	2765.64 ± 744.83	0.85	3135.22 ± 1050.68	2832.21 ± 663.47	0.22

Data presented as mean ± SD. † Mann-Whitney test was used for analysis.
